# Phase IIIb, open-label single-arm trial of darunavir/cobicistat (DRV/COBI): Week 48 subgroup analysis of HIV-1-infected treatment-nave adults

**DOI:** 10.7448/IAS.17.4.19772

**Published:** 2014-11-02

**Authors:** Karen Tashima, Gordon Crofoot, Frank L Tomaka, Thomas N Kakuda, Anne Brochot, Simon Vanveggel, Magda Opsomer, William Garner, Nicolas Margot, Joseph M Custodio, Marshall W Fordyce, Javier Szwarcberg

**Affiliations:** 1 The Miriam Hospital, Providence, RI, USA; 2 Gordon Crofoot Research, Houston, TX, USA; 3 Janssen Research & Development, LLC, Titusville, FL, USA; 4 Janssen Research & Development, LLC, Beerse, Belgium; 5 Janssen Infectious Diseases BVBA, Beerse, Belgium; 6 Gilead Sciences, Foster City, CA, USA

## Abstract

**Introduction:**

COBI, a PK enhancer with no ARV activity is a more selective cytochrome P450 (CYP)3A inhibitor than ritonavir (RTV), does not induce CYP isozymes, and thus has less potential for drug-drug interactions. COBI boosts DRV PK as effectively as RTV in healthy volunteers.

**Materials and Methods:**

This 48-week, phase IIIb, open-label, single-arm, US multicentre study (NCT01440569) included HIV-infected treatment-nave and experienced adults with no DRV RAMs, viral load (VL) ≥1000 c/mL, eGFR ≥80 mL/min and genotypic sensitivity to investigator-selected N[t]RTIs. Patients received DRV/COBI 800/150 mg qd (as single agents) plus two fully active N[t]RTIs. The primary endpoint was any treatment-emergent grade 3 or 4 AEs through Week 24. We report 48-week safety, efficacy and PK/PD results in treatment-nave patients.

**Results:**

Of 313 ITT patients, 295 were treatment-nave (94%). In the treatment-nave cohort, 90% were male, 60% white and 294 (99.7%) received a TDF-containing regimen. Median baseline (BL) VL was 4.8 log_10_ c/mL and CD4^+^ 370 cells/mm^3^. Treatment-emergent grade 3 or 4 AEs regardless of causality were reported in 21 (7%) patients. AEs regardless of causality (any grade; ≥10% of patients) were: diarrhoea (27%), nausea (23%), URTI (15%) and headache (12%). Sixteen (5%) patients had AEs leading to study drug discontinuation, most frequently rash (three patients), hypersensitivity and nausea (two patients each). Consistent with the known inhibition of tubular creatinine secretion by COBI, there was a mean increase from BL in serum creatinine by week 2 (0.09 mg/dL), remaining stable through week 48 (mean 0.10 mg/dL increase from BL). At week 48, 83% of patients achieved VL<50 c/mL; FDA Snapshot); median increase in CD4^+^ was 169 cells/mm^3^. Eight patients met the criteria for resistance testing. M184V was detected in one pt receiving FTC. New primary RAMs were not detected in the other seven patients. The mean population PK-derived DRV AUC_24h_ was 100,620 ng.h/mL and C_0h_ 2,105 ng/mL (n=281). There were no clinically relevant relationships between DRV exposure and virologic response, AEs or laboratory parameters.

**Conclusions:**

The DRV PK of DRV/COBI was consistent with historical data for DRV/RTV. DRV/COBI 800/150 mg qd plus two N(t)RTIs had an 83% response and was well tolerated through Week 48. These results are similar to published data for DRV/RTV 800/100 mg qd, and support the use of DRV/COBI 800/150 mg qd in treatment-nave patients.

